# Association between coronary monosodium urate deposits at DECT and high-risk coronary plaque phenotypes and other features in gout patients

**DOI:** 10.1186/s41747-025-00611-z

**Published:** 2025-08-11

**Authors:** Pietro G. Lacaita, Andrea S. Klauser, Julia Held, David Haschka, Gerlig Widmann, Gudrun M. Feuchtner

**Affiliations:** 1 Department of Radiology, Medical University of Innsbruck, Innsbruck, Austria; 2 Department of Internal Medicine II, Medical University of Innsbruck, Innsbruck, Austria

**Keywords:** Atherosclerosis, Arthritis (gouty), Calcium, Coronary artery disease, Tomography (x-ray computed)

## Abstract

**Background:**

Dual-energy computed tomography (DECT) detects monosodium urate (MSU) deposits in joints. However, the correlation between coronary atherosclerosis phenotypes and MSU-positive lesions in the cardiovascular system remains unclear. We investigated the correlation between coronary MSU-positive plaques on unenhanced DECT with the coronary atherosclerosis profile at coronary CT angiography.

**Methods:**

One hundred fifty rheumatologic patients were prospectively enrolled. Sixty of them underwent unenhanced DECT and 128-row DECT coronary angiography. Analysis included CAD-RADS stenosis severity, high-risk plaque (HRP) phenotypes, and coronary artery calcium (CAC) score.

**Results:**

Of 60 patients, with a mean age of 63.7 years, including 7 females (11.7%), 37 had gout (61.7%), 9 had hyperuricemia (15%), and 14 had other rheumatologic diseases (23.3%). At DECT, 11 (18.3%) had coronary MSU-positive lesions totaling 24 lesions (left anterior descending, 12; right coronary artery, 10; circumflex, 1; left main, 1). HRP phenotypes were identified in 14 of 60 patients (23.3%). The prevalence of HRP was higher in MSU-positive than MSU-negative patients (63.3% *versus* 14.2%; *p* = 0.003; odds ratio 9.91; 95% confidence interval [CI]: 2.30–48.41). CAD-RADS and CAC scores correlated with the number of MSU-positive lesions (ρ = 0.412; 95% CI: 0.167–0.609; *p* < 0.001) and ρ = 0.412; 95% CI: 0.169–0.609; *p* < 0.001). None of the major cardiovascular risk factors (smoking, hypertension, dyslipidemia, or diabetes) was associated with MSU-positive lesions.

**Conclusion:**

We found an association between coronary MSU-positive lesions and HRP-phenotypes, as well as a correlation with stenosis severity and calcium burden. MSU-positive lesions may serve as an unenhanced DECT-derived biomarker of increased cardiovascular risk.

**Relevance statement:**

The detection of coronary MSU-positive lesions by DECT could indicate an increased likelihood of HRP phenotypes. These findings suggest their potential as imaging biomarkers for cardiovascular risk, using unenhanced spectral DECT scans or photon-counting CT.

**Key Points:**

- Identifying gout patients with increased cardiovascular risk remains challenging.
- Coronary MSU-positive lesions detected on unenhanced DECT may be associated with HRP features on coronary computed tomography angiography.
- MSU-positive lesions could serve as biomarkers for cardiovascular risk in gout patients.

**Graphical Abstract:**

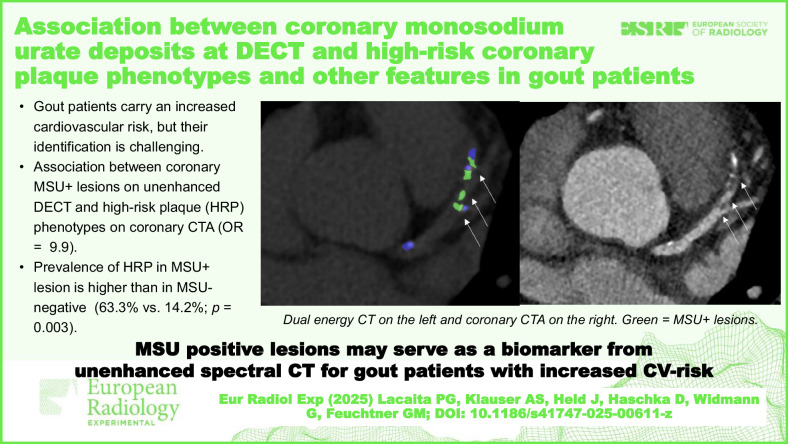

## Background

Gout is a prevalent inflammatory arthritis marked by the deposition of monosodium urate (MSU) crystals within joints and other tissues [1–3]. This condition leads to acute inflammatory responses and, over time, can result in chronic tophaceous gout [4], increasing morbidity and disability-adjusted life-years [5]. Beyond the musculoskeletal system, gout is associated with an elevated risk of cardiovascular diseases, including coronary artery disease (CAD) [6–10].

Chronic inflammation and endothelial dysfunction are central mechanisms underlying this association. A more recent hypothesis suggests that direct MSU detection in coronary vessel walls by dual-energy computed tomography (DECT) [11] may serve as a driver for plaque inflammation and rupture. DECT can distinguish MSU deposits from other substances by utilizing different energy absorption spectra, providing a non-invasive method to assess the extent of MSU crystal deposition [12, 13]. The accuracy of DECT has been validated for identifying MSU deposits in larger structures, such as tophi in peripheral joints [14], as well as for detecting MSU in coronary plaque [15]. However, the morphological correlate in terms of coronary plaque composition of MSU-positive lesions has not been evaluated so far.

Understanding the association between MSU-positive lesions and coronary plaque composition could help identify gout patients and individuals with hyperuricemia who are at a higher risk of adverse cardiovascular events. Whether or not to treat patients with asymptomatic hyperuricemia with regard to cardiovascular risk prevention is a current matter of debate [16]. This study investigates whether coronary MSU deposits detected by DECT can serve as biomarkers for high-risk coronary plaques, potentially aiding in better risk stratification and management of cardiovascular disease in gout patients.

Therefore, the aim of our study was to correlate coronary MSU-positive lesions on DECT with high-risk plaque (HRP) phenotypes, coronary stenosis severity, and coronary artery calcium (CAC) scores using 128-dual source computed tomography angiography (CTA).

## Methods

### Study design, population, and clinical/laboratory data collection

This prospective study was conducted in accordance with the Declaration of Helsinki. Approval of the Institutional Review Board (IRB) (Ethics Commission of the Medical Faculty, Medical University of Innsbruck, Austria; reference no. 1331/2019). Participants gave their written informed consent.

Patients were initially recruited at our university’s rheumatology department following a confirmed clinical diagnosis (see details below) and subsequently referred to the radiology department for DECT, followed or not followed by CTA. Exclusion criteria for CTA were: (1) contraindication for iodinated contrast agent (*e.g*., with estimated glomerular filtration rate < 60 mL/m^2^); and (2) lack of patient consent to iodinated contrast agent administration. Patients were stratified according to their diagnosis as follows: score 1 = gout; score 2 = hyperuricemia (serum uric acid [SUA] level > 6.0 mg/dL); or 3 = other rheumatic diseases (such as rheumatic arthritis and others). Patients were categorized into gout or hyperuricemia groups based on the European League Against Rheumatism’s evidence-based recommendations for diagnosing gout [17]. Then, all patients underwent a three-step computed tomography (CT) protocol as described below. Clinical and laboratory parameters were collected from the patients at the time of recruitment (lipid panels, SUA-level, and others), and established cardiovascular risk factors (dyslipidemia, hypertension, cigarette smoking, diabetes mellitus, and obesity) were also collected [18].

### CT protocol and image analysis

The exams were conducted using a 128-row dual-source CT scanner (Somatom Definition Drive; Siemens Healthineers). A three-step protocol was employed: (1) thoracic DECT; (2) CAC score; and (3) coronary CTA

#### Thoracic DECT

An unenhanced CT scan covering the thoracic range from the aortic arch to the diaphragm was performed in DECT mode and prospective electrocardiography (ECG)-gating in sequential mode. Scan parameters were 2 × 64 × 0.625 mm^3^ at a rotation time of 280 ms. One tube voltage was set at 100 kVp and the other at 140 kVp. The settings were 100 kV/100–140 mAs for tube A and 140 kV/200–250 mAs for tube B. The dual-source DECT scanner simultaneously acquired images at two energy levels using separate x-ray tubes and detectors positioned 90° to 95° apart. Independent tube current modulation and iterative reconstruction were applied. Image reconstruction of 0.75-mm transverse sections with a 0.4 mm overlap was performed in soft tissue kernel (D30) and bone kernel (B60). The D30 kernel was used for dual-energy processing and MSU detection. The data sets were reconstructed using SyngoVIA (Siemens Healthineers) dual-energy software, employing a standardized two-material decomposition algorithm for specific clinical applications. DECT images were reconstructed with a ratio of 1.36, a range of 4, a minimum of 150 HU, and a maximum of 500 HU. The gout algorithm differentiates MSU from calcium using soft tissue as the baseline. This two-material decomposition algorithm is based on the principle that materials with a high atomic number, such as calcium, exhibit higher attenuation increases at higher photon energies compared to materials with low atomic numbers, like MSU. This attenuation difference is independent of the material’s density or concentration [19]. Once separated and characterized, the materials are color-coded and overlaid on multiplanar reformatted cross-sectional images. Green pixels were chosen to represent MSU deposits using the SyngoVIA workstation software (Siemens Healthineers).

The postprocessing software allowed for real-time manipulation of images at source resolution, in any plane, and in 2D and 3D, to best depict MSU deposits. Preprocessed and processed images were transferred to the picture archiving system. Corresponding preprocessed grayscale images were reviewed for deposits [20]. The plaque volume was calculated using the SyngoVIA workstation software (Siemens Healthineers). The regions of interest (ROIs) were identified and calculated automatically by the software and subsequently evaluated by two independent radiologists to ensure accuracy and consistency.

Localization of calcified plaques and MSU deposits for the coronary arteries was assigned to the right coronary artery, left main artery, circumflex artery, and left anterior descending artery. Small (< 3 mm) green pixels adjacent to calcifications were defined as artefactual and not scored to avoid artifacts falsely mimicking MSU deposits, as well as blurry artifacts were also excluded. According to American College of Radiology/European League Against Rheumatism guidelines, nail bed deposits, submillimeter deposits, skin deposits, and deposits obscured by motion, beam hardening, vascular artifacts, rib cartilage artifacts, and bronchial calcifications were not classified as positive findings in our study [21, 22].

#### CAC score

An unenhanced ECG-gated scan with standardized scan parameters (detector collimation 2 × 64 × 0.6 mm^3^; 120 kVp; image reconstruction 3-mm slice width, increment 1.5 mm) and prospective ECG-triggering in dual-source high-pitch mode was performed. The CAC score in Agatston units [23] of all coronary arteries was calculated with automated software (Cardiac CT, SyngoVIA, Siemens Healthineers).

#### Coronary CTA

It was performed by using a 128-row dual-source CT (Definition FLASH or DRIVE, Siemens Healthineers) with a detector collimation of 2 × 64 × 0.6 mm^3^ and a rotation time of 0.28 s, acquiring 128 slices with *z*-flying spot. Scans were triggered into the arterial phase using bolus tracking (threshold of 100 HU in the ascending aorta). An iodinated contrast agent was injected (Iopromide, Ultravist 370™, Bayer Healthcare) into an antecubital vein using an automated injector. The contrast volume was calculated using a standardized regimen, based on the patient´s body weight. Contrast volume and flow rate (4 mL/s, 5 mL/s, or 6 mL/s) were adjusted according to the patient’s weight. The contrast volume ranged from 60 mL to 110 mL. Prospective ECG-triggering (< 65 beats per min) or retrospective ECG-gating (> 65 beats per min or in cases of arrhythmia, including atrial fibrillation and other rhythm abnormalities such as premature beats that could affect image quality), was performed depending on heart rate. Tube voltage ranged from 80 kVp to 140 kVp, and was adjusted to the patients´ body mass and dimensions using an automated tool (CARE kV^TM^, Siemens Healthineers). Similarly, tube current (mAs) was adapted automatically (CARE Dose4D^TM^, Siemens Healthineers).

Patients received betablockers if the baseline heart rate was above 80 beats per min, and optionally, if above 65 beats per min. Five milliliters of Metoprolol (Beloc^TM^, AOP Orphan Pharmaceuticals GmbH) was injected intravenously into an antecubital vein, and repeated after 5 min, if necessary. Oral premedication with betablockers was recommended to referring physicians 1 h prior to the exam, if possible.

Axial thin-slice images at best diastolic and systolic phases were reconstructed with a 0.75-mm slice width (increment, 0.4 mm) and transferred to a three-dimensional postprocessing software (CT Cardiac; SyngoVIA, Siemens Healthineers).

Curved and oblique interactive multiplanar reformations using a client-server-based software (SyngoVia^TM^, Siemens Healthineers) were generated, and the following outcome measures were evaluated:

- Coronary stenosis severity—scored visually according to CAD-RADS^TM^ [24] score (0–5) as minimal (1) < 25%, mild (2) 25–49.9%, moderate (3) 50–69.9%, severe (4) ≥ 70–99%, and (5) occluded 100% on a per-coronary segment-base (American Heart Association-modified-17-segment classification), assisted by quantitative stenosis measurement using curved multiplanar reformations;
- Coronary plaque phenotypes—HRP analysis was performed according to the CAD-RADS/HRP criteria [24]. Low attenuation plaque (LAP) was defined as a hypoattenuating lesion with < 150 HU. CT density was screened with the “pixel lens” and the lowest HU recorded [25]. LAP < 30 HU was defined as lipid-rich necrotic core [26], and LAP < 60 HU as fibrofatty. Napkin-ring sign was defined as an outer high-density rim with an inner hypodense area [27]. Spotty calcification (SC) was defined as a calcification of less than 3-mm in size. Positive remodeling was defined as a remodeling index of > 1.1 [28]. An HRP was defined if a minimum of two criteria were present, and if at least one LAP < 30 HU or LAP < 60 HU was present per patient.

Coronary CTA analysis and Gout DECT analysis were performed by one highly experienced reader (> 10-years of experience in cardiac CT) as part of clinical standardized reporting.

### Statistical analysis

Statistical analysis was performed using SPSS™ software (V25.0, SPSS Inc.). Quantitative variables are expressed as means ± standard deviation or as median (interquartile range), according to their distribution, and categorical variables as absolute values and percentages. The χ^2^ test was applied to test for differences in categorical data. The correlation of MSU lesions with CAC score and CAD-RADS was defined by Spearman's rank correlation coefficient (ρ).

Data analysis involved the use of crosstabs to compare coronary MSU-positive and MSU-negative groups regarding HRP, stenosis severity, and CAC scores. Odds ratios (ORs) and 95% confidence intervals (CIs) were calculated to quantify the strength of associations between MSU-positive lesions and coronary plaque phenotypes. Univariate and multivariate binary regression analysis was performed for the association of MSU-positive lesions with HRP and the major cardiovascular risk factors, and the ORs were calculated.

## Results

### Patients’ demographics and baseline characteristics

**Fig. 1:**
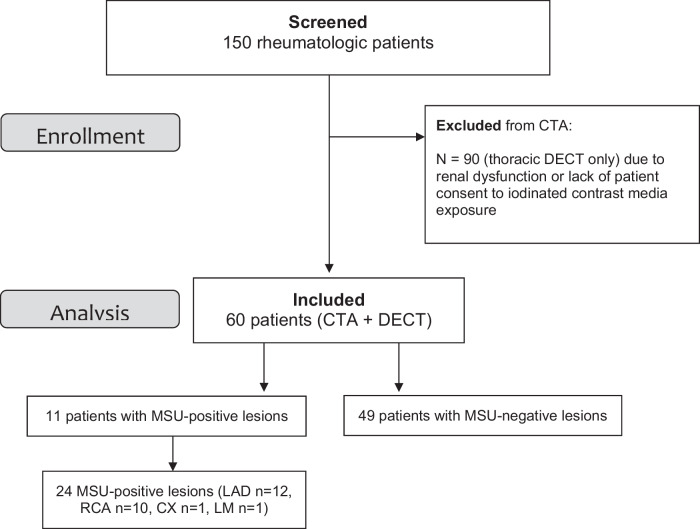
Study flowchart. CX, Circumflexcoronary artery; LAD, Left anterior descending; LM, Left main coronary artery; RCA, Right coronary artery

Of 150 rheumatologic patients enrolled, 90 did not undergo CTA due to exclusion criteria (lack of patient consent to iodinated contrast administration or contraindications to iodinated contrast agent, such as renal dysfunction) and underwent unenhanced thoracic DECT only. Patient enrollment is illustrated in Fig. 1. Of 60 patients who underwent both thoracic DECT and coronary CTA, one patient had a prior percutaneous stent implantation. Age of the study population was 63.7 ± 8.09 years (mean ± standard deviation), ranging from 59 to 79 years, and 7 (11.7%) were females; 37 (61%) had gout, 9 (15%) hyperuricemia, and 14 (23.3%) other rheumatologic diseases, such as rheumatic arthritis, Still/Sjögren syndrome, and others.

Regarding cardiovascular risk factors, the prevalence of active cigarette smoking was 15%, while a relevant proportion exhibited hypertension (35%) and dyslipidemia (38.3%). Table 1 shows the study cohort profile, their clinical diagnosis, major cardiovascular risk factors, laboratory values (SUA), and chest pain complaints.

**Table 1:** Study cohort (*n* = 60)

Age (years)	63.7 ± 8.09
Females	7 (11.7%)
Body mass index (kg/cm^2^)	25.2 ± 8.7
Active smoking	9 (15%)
Positive family history	15 (25%)
Arterial hypertension	21 (35%)
Dyslipidemia	23 (38.3%)
Diabetes	7 (11.7%)
Clinical diagnosis	
Gout	37 (61.7%)
Hyperuricemia	9 (15%)
Other rheumatologic diseases	14 (23.3%)
SUA (mg/dL)	6.11 ± 1.74
Typical chest pain	5 (8.3%)
Dyspnea	7 (11.7%)
No chest pain	48 (80%)

Data are given as mean ± standard deviation or as number (percentage) *SUA* Serum uric acid

### DECT findings

**Fig. 2:**
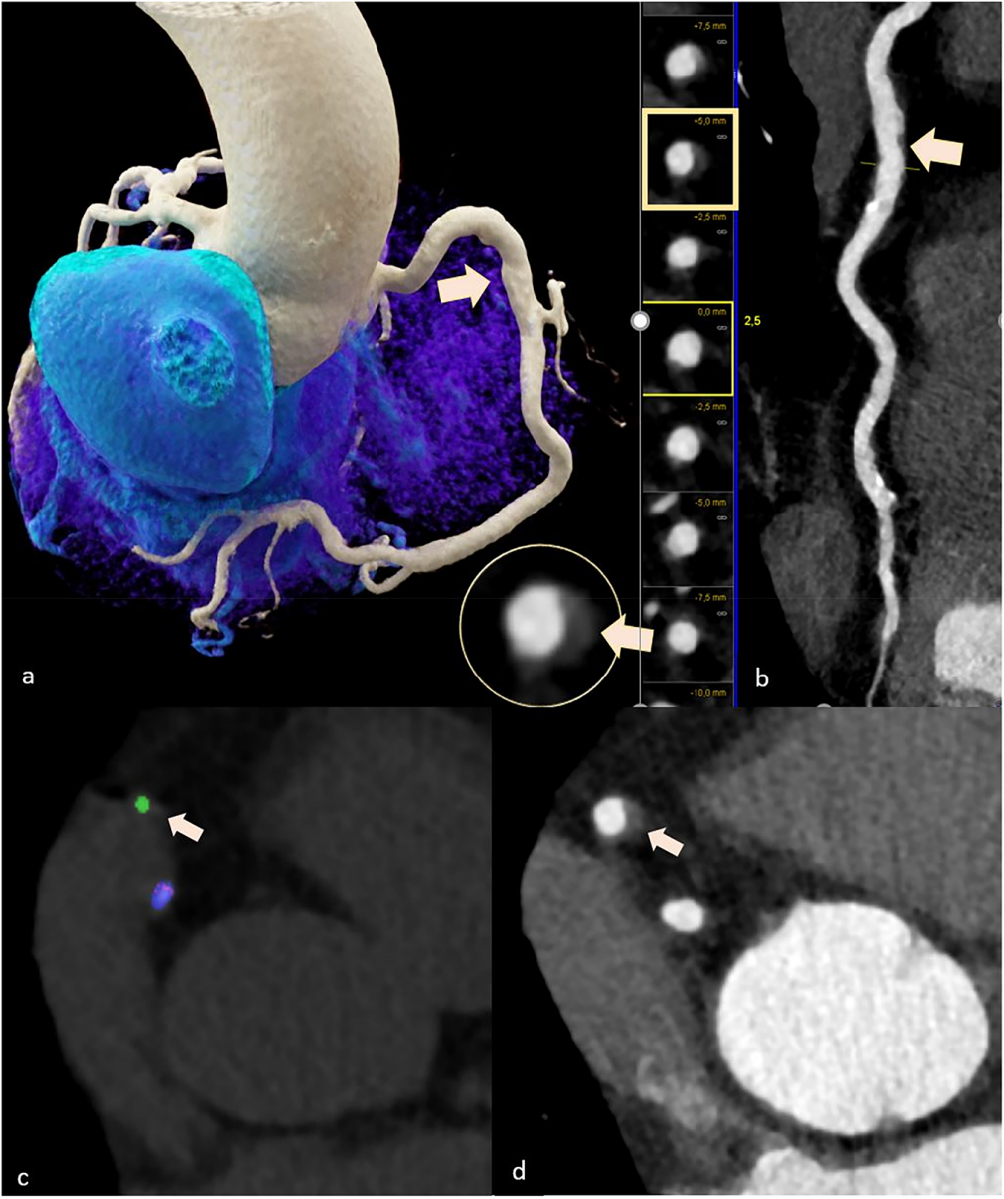
A 63-year-old male with gout and a high-risk low attenuation plaque (28–50 HU and positive remodeling) (arrows in **a**, **b**, and **d**) in the proximal RCA, which correlated with an MSU-positive lesion (arrow in **c**). Three-dimensional volume rendering technique (**a**) and curved multiplanar reconstruction (**b**). DECT scan (**c**). **d** Coaxial CTA image alignment of the proximal RCA (**d**). Green = MSU-positive lesion. In this patient, the CAC score was 565 Agatston units. CAC, Coronary artery calcium; DECT, Dual-energy computed tomography; MSU, Monosodium urate; RCA, Right coronary artery

**Fig. 3:**
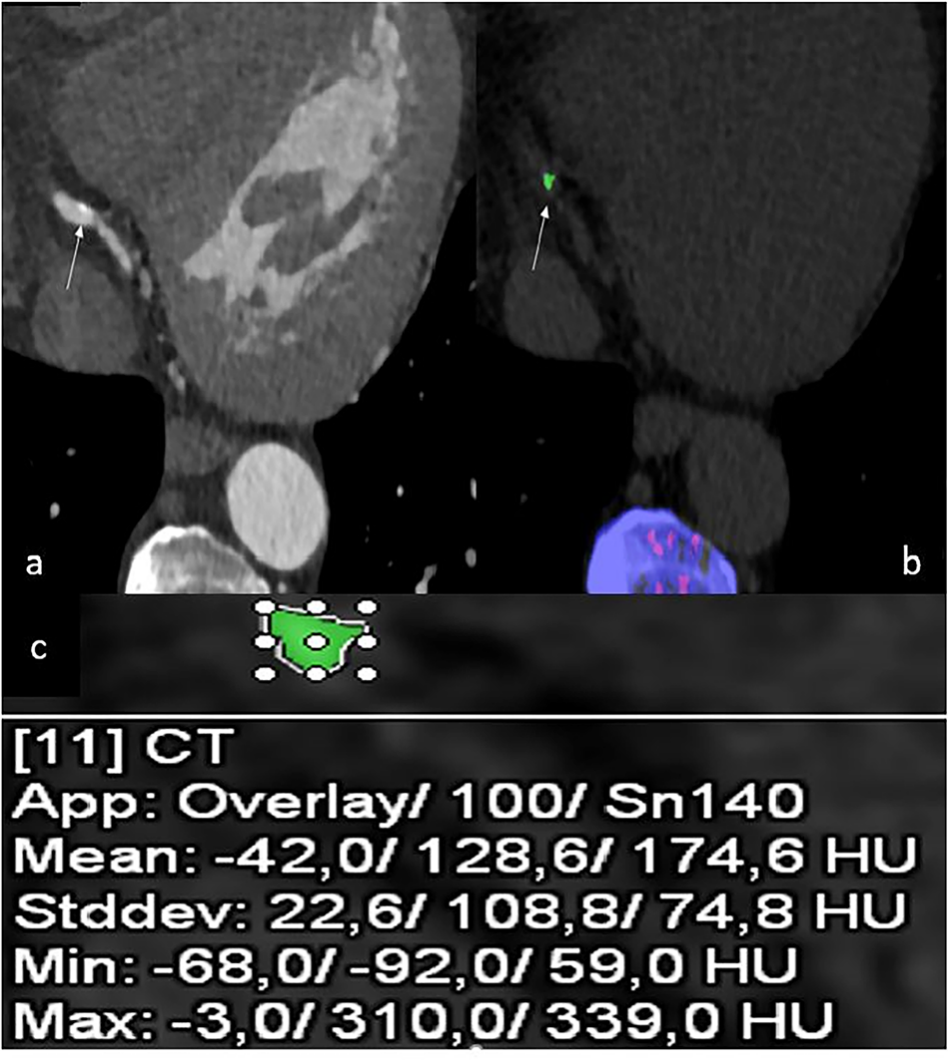
The same 63-year-old male patient with gout presented in Fig. 1, here shown for a calcified plaque in the distal RCA (arrow in **a**), also correlated with an MSU-positive lesion (arrow in **b**). Coronary CTA axial view (**a**) and DECT scan (**b**). Calculated density and volume of the MSU-positive lesions (**c**). Green = MSU-positive lesion. CTA, Computed tomography angiography; DECT, Dual-energy computed tomography; MSU, Monosodium urate; RCA, Right coronary artery

**Fig. 4:**
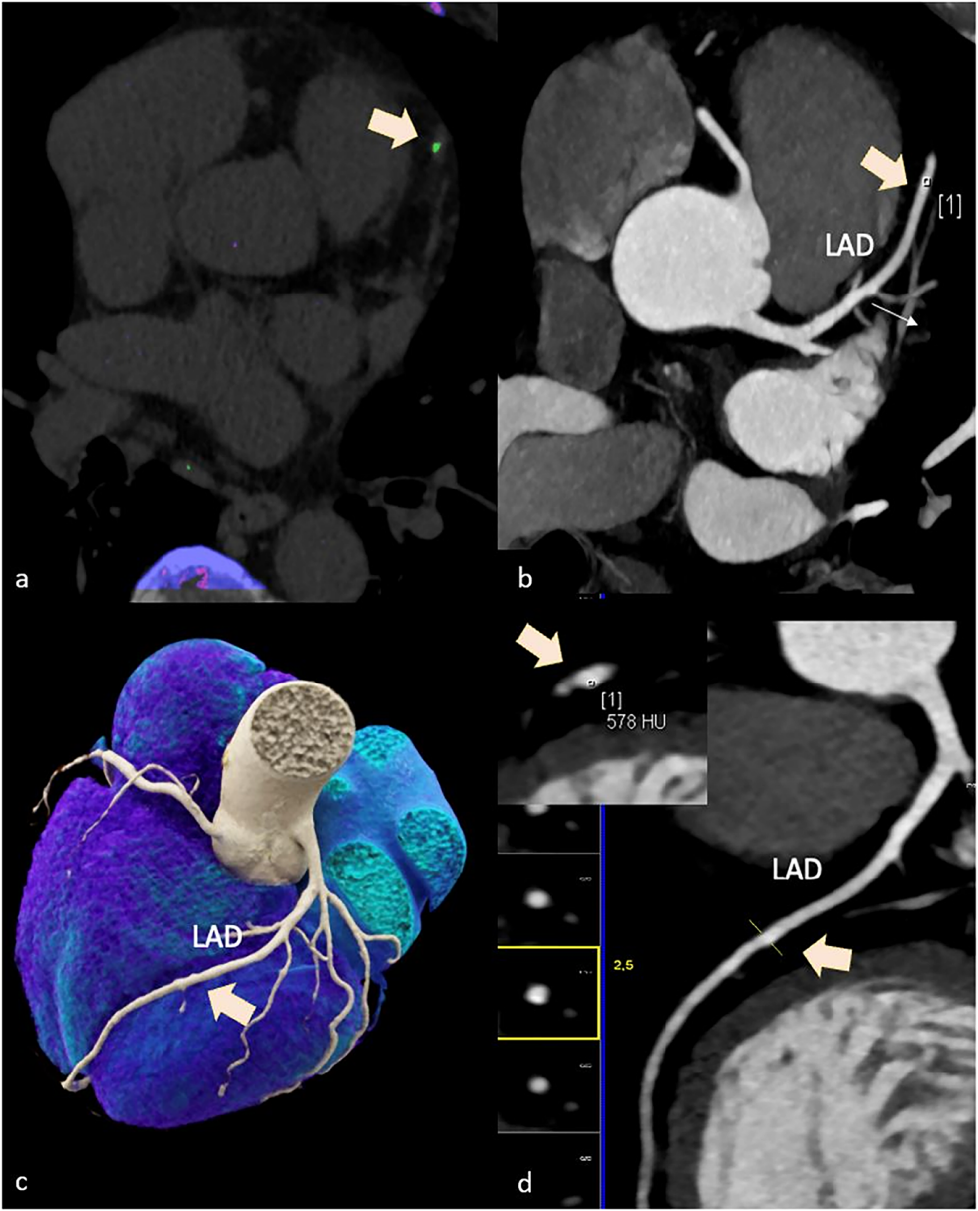
A 74-year-old male with gout (**a**) and a small coronary plaque with an equivalent CT density (578 HU) to the coronary lumen (arrow in **b**) in the distal LAD artery, which was difficult to visualize and correlated with an MSU-positive lesion on DECT (arrow in **a**). Green = MSU-positive lesion on DECT scan (**a**); coronary CTA (**b**) Three-dimensional volume rendering technique (**c**) and curved multiplanar reconstruction (**d**). In this patient, the CAC score was minimal with 3.4 Agatston units. CAC, Coronary calcium; CTA, Computed tomography angiography; DECT, Dual-energy computed tomography; LAD, Left ascending coronary artery; MSU, Monosodium urate

**Fig. 5:**
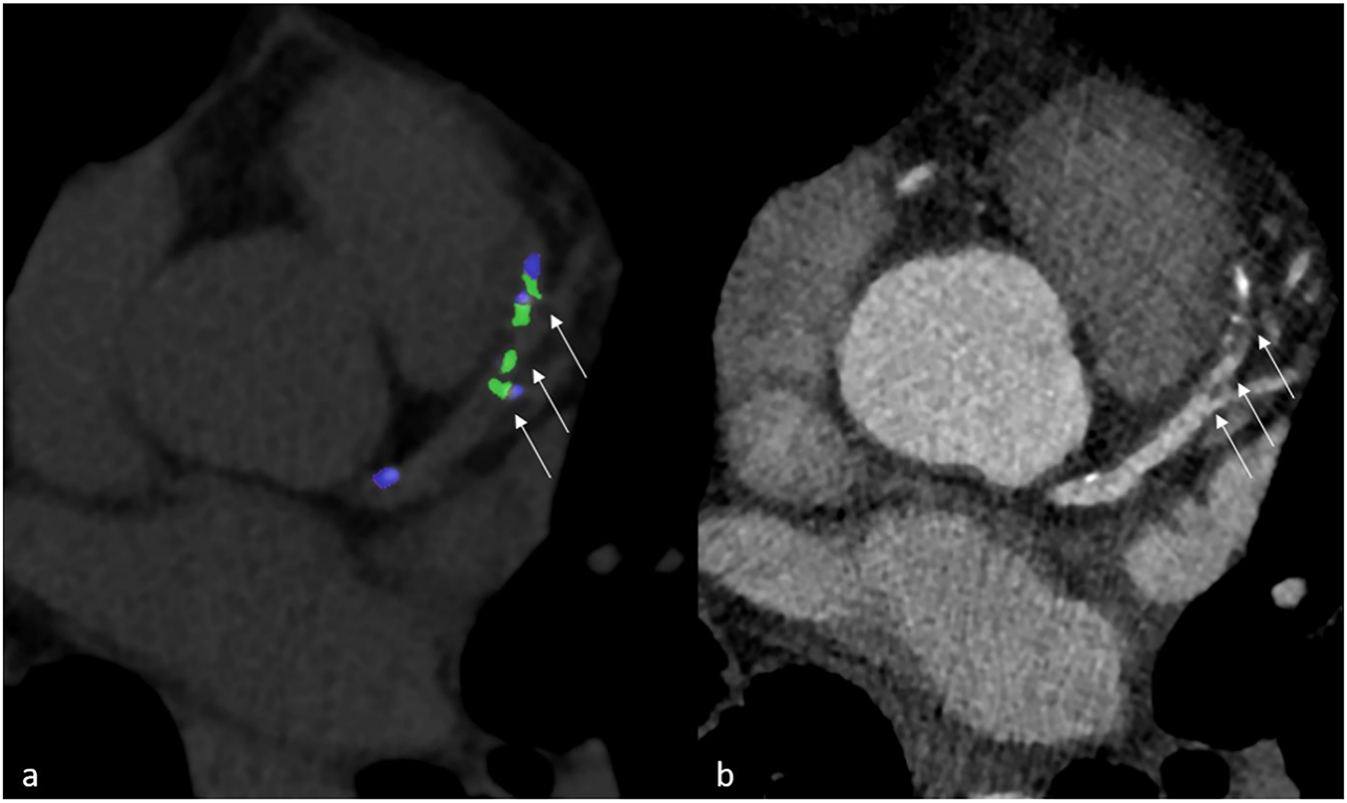
A 75-year-old male with severe tophaceous gout and mixed plaque in the mid LAD coronary artery with HRP criteria. **a** DECT scan with multiple MSU-positive lesions (arrows). **b** Coronary CTA showing a high-risk plaque (HRP) with low-attenuation components (mean 57 HU) and positive remodeling (arrows). Blue on DECT (**a**) calcified coronary plaque

Of the 60 patients included, 11 (18.3%) had coronary MSU-positive lesions (24 lesions in total: left anterior descending artery, *n* = 12; right coronary artery, *n* = 10; circumflex artery, *n* = 1; left main artery, *n* = 1). HRP phenotypes were identified in 14 of 60 patients (23.3%). Mean CT density of the MSU-positive lesions was 195.6 ± 129.0 (mean ± standard deviation), ranging from 100 HU to 634 HU, and plaque volume was 10.9 mm^3^ ±13.9 (mean ± standard deviation), ranging from 1.0 to 60.4. Figures 2–5 show case examples illustrating the correlation of CTA with DECT findings.

### Association between coronary MSU-positive lesions and HRP

Among the coronary MSU-positive patients, 7/11 (63.6%) had HRP, compared to 7 of 49 (14.2%) MSU-negative patients. The prevalence of HRP was higher in MSU-positive than MSU-negative patients (63.3% *versus* 14.2%; *p* = 0.003; OR 9.91; 95% CI: 2.30–48.41). Mean density of the LAP component of the HRP was 43.1 ± 21.4 (mean ± standard deviation).

### Association between coronary MSU-positive lesions with CAD-RADS and CAC scores

CAD-RADS and CAC scores were correlated with the number of MSU-positive lesions (*r* = 0.412; *p* < 0.001; 95% CI: 0.167–0.609) and *r* = 0.412; *p* < 0.001, 95% CI: 0.169–0.609), respectively.

**Fig. 6:**
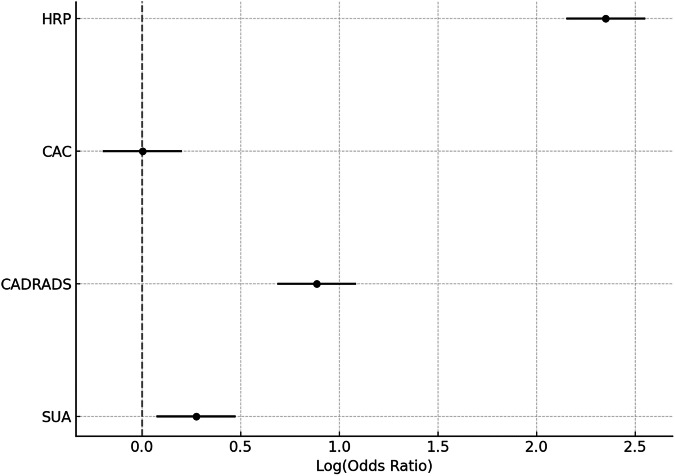
Forest plot illustrating the association of CTA parameters for HR, CAC score, coronary stenosis severity (CAD-RADS), and the SUA-level in patients with MSU-positive lesions on: DECT, Dual-energy computed tomography; CAC, Coronary artery calcium score; CAD-RADS, Coronary Artery Disease Reporting and Data System; CTA, Computed tomography angiography; HRP, High-risk plaque; MSU, Monosodium urate; SUA, Serum uric acid

Univariate binary regression analysis (Table 2) showed an association of coronary MSU-positive lesions with HRP (OR 10.50, 95% CI: 2.42–45.55; *p* = 0.002), and a lower association with the CAC score (OR 1.003, 95% CI: 1.000–1.005; *p* = 0.019). The CAD-RADS score was also associated with coronary MSU-positive lesions (OR 1.32, 95% CI: 1.33–4.4; *p* = 0.026) (Fig. 6).

**Table 2:** Univariate binary regression analysis for prediction of MSU-positive lesions on DECT

	OR	95% CI	*p*-value
High-risk plaque	10.5	2.424–45.488	0.002
CAC	1.003	1.000–1.005	0.019
CAD-RADS	2.425	1.331–4.419	0.004
SUA level	1.316	0.890–1.948	0.167
Active smoking	0.229	0.258–2.273	0.630
Arterial hypertension	0.738	0.169–3.216	0.686
Family history	2.242	0.535–9.399	0.269
Dyslipidemia	1.053	0.262–4.224	0.942
Diabetes	2.200	0.362–13.370	0.392

The table shows an association between MSU-positive lesions (per patient) and HRP, CAC, and coronary stenosis severity (CAD-RADS). However, none of the other major cardiovascular risk factors (CVRF) were associated with MSU-positive lesions

*CAC* Coronary artery calcium score, *CAD-RADS* Coronary Artery Disease Reporting and Data System, *SUA* Serum uric acid

None of the other major cardiovascular risk factors (active and former smoking, arterial hypertension, positive family history, dyslipidaemia, diabetes) nor SUA level (OR 1.32, 95% CI: 0.90–1.95, *p* = 0.167), were associated with MSU-positive lesions on DECT.

## Discussion

The significant association between coronary MSU-positive lesions and HRP phenotypes underscores the potential impact of gout as a driver for CAD progression. Patients with coronary MSU-positive lesions are approximately ten times more likely to present with HRP compared to their MSU-negative counterparts. This finding aligns with the hypothesis that the chronic inflammatory environment induced by MSU crystals contributes to plaque vulnerability. HRP Criteria are novel CTA imaging biomarkers predicting cardiovascular events [26], with a four-fold increased risk of events, independent of coronary stenosis severity. Coronary stenosis severity is also an important predictor of cardiovascular outcomes, because cardiovascular risk increases along with coronary stenosis severity. The major HRP criterion is the low-attenuation plaque (LAP) with a CT density of less than 30 HU, a surrogate for lipid-rich necrotic core plaque, carrying the highest risk for rupture and major adverse cardiac events [26]. Other HRP criteria include positive remodeling, spotty calcification, and the napkin ring sign [24–28].

Further, we identified a moderate correlation between coronary MSU-positive lesions and both stenosis severity (*r* = 0.412) and CAC scores *r* = 0.412). This indicates that coronary MSU-positive lesions are more closely linked to plaque characteristics indicative of instability rather than the extent of luminal narrowing or calcification. These findings suggest that MSU deposition may be contributing not only to plaque instability but also to coronary artery calcification, underscoring the multifaceted role of gout in cardiovascular disease.

Further, we found a correlation of coronary MSU-positive lesions with stenosis severity and CAC scores, while the OR for HRP was higher than for CAD-RADS and CAC, indicating that MSU positivity may be more closely linked to adverse plaque characteristics indicative of instability rather than the extent of luminal narrowing or the CAC burden. This highlights the need for comprehensive plaque assessment beyond traditional measures of stenosis and calcified burden in gout patients. Consistent with epidemiological studies showing increased cardiovascular events in gout patients, our findings provide a potential mechanistic explanation for this elevated risk.

The chronic inflammation associated with persistent hyperuricemia and MSU deposition likely contributes to endothelial dysfunction, plaque formation, and instability [29]. In gout patients, persistent hyperuricemia leads to the deposition of MSU crystals in joints and tissues, triggering local and systemic inflammatory responses. Inflammatory cytokines and chemokines, triggered by MSU crystals, promote oxidative stress and reduce nitric oxide availability, leading to endothelial dysfunction. This dysfunction facilitates the migration of inflammatory cells into the arterial wall, contributing to atherosclerosis and plaque vulnerability. The possible role of MSU deposits in plaque instability underscores the need to investigate dual-energy CTA-based imaging biomarkers, which may improve the precision of gout patient management.

While our study provides important insights, it also raises questions about whether lowering serum uric acid (SUA) levels can mitigate cardiovascular risk in this population. Therapies that can effectively modulate the inflammatory cascade triggered by MSU crystals may play a crucial role in preventing plaque rupture and subsequent cardiovascular events. However, SUA levels are often highly variable in longitudinal measurement series, limiting their accuracy as potent predictor of cardiovascular risk, while the direct visualization of MSU crystals in the cardiovascular system by DECT may more accurately predict higher cardiovascular risk. Further, we excluded lesions representing more likely artifacts based on their appearance (*e.g*., if motion blurring or other artifacts were present), and those with a smaller size of less than 3 mm, due to their higher likelihood of artifacts [30]. Long-term follow-up studies are required to assess whether these lesions are true artifacts or may impact cardiovascular risk stratification. Finally, of note, we used dual-source CT technology for dual-energy subtraction. Recently introduced photon-counting CT [31] provides improved spectral imaging capability at a higher spatial resolution, making the technology applicable in clinical practice without adding additional contrast agents and radiation exposure.

Despite the interesting findings, this study has several limitations to be acknowledged. First, the sample size is relatively small, which may limit the generalizability of the results and statistical power. In particular, some 95% CIs were large due to the small sample size. Second, the cross-sectional design of the study precludes any causal inferences. Third, while DECT may detect coronary MSU deposits, it may not capture all the complexities of plaque morphology and composition, and the lesions detected appear larger than MSU crystals in atherosclerotic plaque, suggesting that they are embedded in other plaque components, such as fibro-fatty tissue. Nonetheless, a coronary MSU-positive lesion from an unenhanced scan could indicate plaque vulnerability, with adherent implications for clinical management of patients with regard to primary preventive measures. Combining DECT with other imaging modalities, such as intravascular ultrasound or optical coherence tomography, might provide a more comprehensive assessment of plaque characteristics. Fourth, the study cohort includes both gout patients, and patients with hyperuricemia without a clinical diagnosis of gout, and other rheumatologic diseases. Finally, given the cross-sectional nature of this study, we were not able to demonstrate direct causality between MSU-positive deposits and CAD progression. Prospective longitudinal studies are needed to assess whether MSU-positive deposits predict CAD progression and whether urate-lowering therapy impacts coronary plaque vulnerability.

Future research should focus on larger cohorts to validate these findings and explore the underlying mechanisms linking MSU deposits to coronary plaque instability. Understanding these mechanisms may pave the way for targeted therapies aimed at reducing cardiovascular risk in gout patients. Additionally, longitudinal studies would be required to assess the impact of coronary MSU-positive lesions on long-term cardiovascular outcomes in gout patients. Exploring the potential benefits of urate-lowering therapies, such as allopurinol or febuxostat, in reducing the prevalence of coronary MSU-positive lesions and HRPs could provide valuable insights into the management of cardiovascular risk in gout patients. Moreover, investigating the role of lifestyle modifications, such as dietary changes, on coronary MSU plaque development might be of interest.

In conclusion, our study indicates a relationship between coronary MSU-positive lesions and high-risk coronary plaque phenotypes in gout patients. The correlations with both features of plaque vulnerability, coronary stenosis severity, and coronary artery calcification suggest that coronary MSU depositions may indicate a higher cardiovascular risk and may act as an imaging biomarker. Incorporating advanced imaging techniques, such as DECT or photon-counting CT, into the routine cardiovascular risk assessment of gout and hyperuricemia patients could aid in the early identification of those at heightened risk of adverse cardiovascular outcomes and prompt primary preventive measures with regards to the management of cardiovascular risk factors and the implementation of targeted therapies to mitigate this risk. Future research is required to explore the predictive value of coronary MSU-positive lesions and their value in clinical practice in gout, hyperuricemia, and other patients.

## Data Availability

The datasets generated and/or analyzed during the current study are not publicly available but are available from the corresponding author on reasonable request.

## Abbreviations

CAC: Coronary artery calcium
CAD: Coronary artery disease
CAD-RADS: Coronary artery disease reporting and data system
CI: Confidence interval
CT: Computed tomography
CTA: Computed tomography angiography
DECT: Dual-energy computed tomography
ECG: Electrocardiography
HRP: High-risk plaque
LAP: Low-attenuation plaque
MSU: Monosodium urate
OR: Odds ratio
SUA: Serum uric acid
